# The relationship between physical exercise and depression among college students: the chain mediating role of self-efficacy and self-identity

**DOI:** 10.3389/fpsyg.2026.1751461

**Published:** 2026-05-08

**Authors:** Xiaolong Zhang, Weijie Gao, Ziyi Zhang, Shan Huang, Kexiang Yang

**Affiliations:** 1 Department of Physical Education, Jeonbuk National University, Jeonju-si, Jeollabuk-do, Republic of Korea; 2 Shaanxi Technical College of Finance and Economics, Xianyang, China; 3 College of Physical Education, China University of Geosciences (Wuhan), Wuhan, China

**Keywords:** college students, depression, physical exercise, self-efficacy, self-identity

## Abstract

**Objective:**

Depression is a common psychological problem among college students, which seriously threatens their physical and mental health and overall development. This study aims to examine the association between physical exercise and depression among college students, and to analyze the mediating associations of self-efficacy and self-identity in this relationship.

**Methods:**

Convenience sampling was adopted to conduct a questionnaire survey among 2,021 college students (36.86% males, 63.14% females; mean age 19.02 ± 1.38 years) from 8 universities in Henan Province. Data were collected using the Physical Activity Rating Scale-3 (PARS-3), the Center for Epidemiologic Studies Depression Scale (CES-D), the General Self-Efficacy Scale (GSES), and the Self-Identity Scale (SIS). Descriptive statistics, correlation analysis, and chain mediating effect analysis were conducted using SPSS 27.0 and the PROCESS 4.2 macro program.

**Results:**

There were significant pairwise correlations among physical exercise, self-efficacy, self-identity, and depression (*p* < 0.01). The indirect association estimates for self-efficacy and self-identity were −0.015 and −0.072, respectively, and the sequential indirect association estimate for the path self-efficacy → self-identity was −0.037. The three indirect association estimates accounted for 6.73%, 32.29%, and 16.59% of the total association, respectively.

**Conclusion:**

Physical exercise was negatively related to depression among college students. In addition, this association was linked to indirect pathways involving self-efficacy, self-identity, and their sequential pathway.

## 1 Introduction

Depression refers to an emotional and mental disorder characterized by persistent low mood (Monteggia et al., 2014). College students are in the transition stage from late adolescence to early adulthood, with relatively immature psychological development. Facing multiple challenges including academic pressure, interpersonal adaptation, and self-cognition restructuring, they may be particularly vulnerable to depressive symptoms. A meta-analysis showed that the detection rate of depressive symptoms among college students worldwide is 25% (Sheldon et al., 2021), and the estimated proportion among Chinese college students reaches 28.4% (Gao et al., 2020). This high prevalence has greatly affected the well-being and overall development of college students. It may negatively influence their academic performance and contribute to negative cognitions such as emotional distress and self-denial. In extreme cases, it may lead to serious consequences such as self-harm, bringing multiple burdens to individuals, families, and society (Gilbert, 2017). Therefore, exploring factors associated with depression among college students may provide useful evidence for mental health education in university settings.

Physical exercise, as an active and healthy lifestyle, has been widely associated with better physical and mental health (Zhang and Min, 2022). Several studies have discussed possible mechanisms underlying the association between physical exercise and lower depressive symptoms. For example, some studies have indicated that physical exercise may be related to the release of endorphins and the regulation of neurotransmitters such as serotonin and dopamine (Hossain et al., 2024). These physiological processes may be associated with lower stress responses, less physical tension, and better mood regulation (Zhao, 2024). In view of the current high incidence of depression among college students, existing studies have further shown that physical exercise is associated with lower levels of emotional problems such as depression and anxiety among college students (Liu et al., 2024; Wei et al., 2024), and may also be related to lower levels of suicidal ideation and suicidal behaviors (Zhang et al., 2022). Based on this, Hypothesis H1 is proposed: Physical exercise is negatively associated with depression among college students.

Self-efficacy refers to an individual’s belief in their ability to successfully complete tasks and achieve goals (Bandura, 1977). Its core value lies in helping individuals maintain a high level of self-confidence and sense of situational control when facing new challenges or difficulties, thereby more effectively managing negative emotions (Tang et al., 2010). According to social cognitive theory, individuals’ self-efficacy stems from direct experiences in life practice. As a structured behavioral practice, physical exercise may provide individuals with continuous goal challenges and successful experiences (Erickson et al., 2012). This positive feedback may strengthen individuals’ beliefs in their own abilities, thereby enhancing self-efficacy (Dong et al., 2023). Existing empirical studies have also confirmed that regular physical exercise is significantly positively correlated with self-efficacy among college students (Wang et al., 2022), and it has also been identified as a possible mediating factor between physical exercise and mental health, suggesting an association pathway of “physical exercise–self-efficacy–mental health” (Raggiotto and Scarpi, 2020). Specifically, through approaches such as goal achievement experiences and distraction from negative thoughts, daily physical exercise may be associated with higher self-efficacy and a stronger sense of situational control, which in turn may be related to lower levels of anxiety, depression, and stress symptoms (Mikkelsen et al., 2017). From the perspective of emotional coping logic, individuals inevitably experience negative emotions when encountering difficulties. However, individuals with low self-efficacy often lack confidence in effective coping and are more inclined to adopt negative avoidance or emotion-focused coping styles (Jex et al., 2001). Such coping styles may exacerbate the accumulation of negative emotions and have an adverse impact on mental health (Medrano et al., 2016). On the contrary, individuals with high self-efficacy usually have more positive self-cognition and behavioral beliefs, and are better at taking the initiative to seek solutions when facing problems, which may be related to fewer depressive symptoms. Therefore, self-efficacy may be regarded as a psychological factor associated with depression among college students (Yuan et al., 2024). Based on this, this study proposes Hypothesis H2: Self-efficacy plays a mediating role between physical exercise and depression among college students.

In addition to self-efficacy, physical exercise may also be associated with the development of self-identity (Cao and Luo, 2024; Yao et al., 2025). Self-identity, also known as ego identity, refers to the continuous and consistent subjective experience and cognition formed by individuals regarding their internal abilities, cognitive beliefs, developmental status, and other aspects (Ochse and Plug, 1986). According to Erikson’s theory of personality development (Erikson, 1968), adolescence and early adulthood are critical periods for establishing self-identity. During this stage, physical exercise can, on the one hand, help individuals gain positive experiences of self-breakthrough by improving physical fitness and refining sports skills, which may be related to higher levels of self-identity (Alfermann and Stoll, 2000). On the other hand, continuous participation in physical exercise enables individuals to clearly recognize their own abilities and values, further enhancing self-acceptance and self-cognition, which may contribute to more positive self-cognition (Piko and Keresztes, 2006). It is worth noting that if an individual fails to establish a stable self-identity, they are prone to falling into an identity crisis. When facing various conflicts, such as academic and interpersonal conflicts, it is difficult for them to form effective coping strategies, which in turn may be associated with greater vulnerability to depressive symptoms. Although there are not many special studies directly exploring the relationship between self-identity and depression among college students, existing limited studies have revealed the association between the two. Research has shown that a stable self-identity is associated with weaker adverse associations between academic stress and mental health (Li et al., 2022) and is associated with a lower likelihood of depression (Cao et al., 2025). In addition, adolescents with an extroverted thinking style towards their families are more likely to establish self-identity by meeting the expectations of family members, actively respond to identity confusion, and thus have a lower incidence of depressive symptoms (Li et al., 2018). Based on this, this study proposes Hypothesis H3: Self-identity plays a mediating role between physical exercise and depression among college students.

Self-efficacy and self-identity are not isolated; they exhibit a clear internal connection and transmission mechanism. Existing studies have confirmed that high self-identity stems from the confirmation of individuals’ positive existence, and self-efficacy, as an individual’s confidence and belief in their own abilities, just provides core support for this positive self-cognition and is significantly positively associated with self-identity (Liu and Cheng, 2017). On the contrary, if an individual has low self-efficacy, their sense of self-control when facing difficulties may be associated with weaker self-control, lower self-identity, and poorer mental health (Yu and Jin, 2022). Therefore, self-efficacy is not only affected by physical exercise but also may be related to higher levels of self-identity. Individuals with high self-efficacy are more likely to gain positive experiences in practice, thereby strengthening the recognition and acceptance of self-worth and reducing the intrusion of negative emotions such as depression and anxiety. Based on this, this study proposes Hypothesis H4: Self-efficacy and self-identity play a chain mediating role in the relationship between physical exercise and depression among college students.

**Figure 1:**
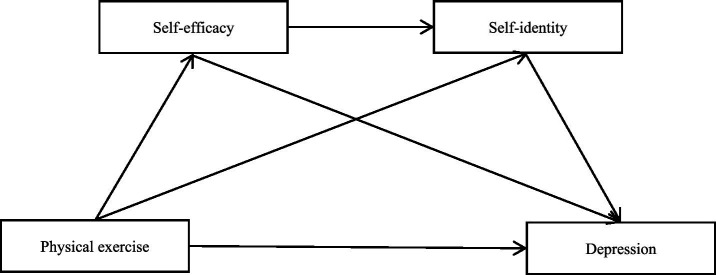
The proposed theoretical model.

In summary, existing literature has consistently reported the negative correlation between physical exercise and depression among college students, and has separately suggested possible mediating associations of self-efficacy and self-identity in this relationship. However, previous studies have largely overlooked the theoretical connection between self-efficacy and self-identity. Based on social cognitive theory, self-efficacy, as a type of belief in ability, may provide the motivational basis for individuals to pursue self-exploration, while self-identity is the psychological outcome of such exploration. This indicates that there may be a sequential relationship between the two rather than mutual independence. Secondly, no previous research has empirically tested whether the sequential chain of “physical exercise → self-efficacy → self-identity → depression” may serve as an indirect association pathway linking physical exercise to depression. Based on this, by integrating these two theoretically related self-related constructs into a unified chain model, this study aims to examine a possible multi-step associative pathway underlying the relationship between physical exercise and depression, thereby providing a more comprehensive theoretical framework for understanding the relationship between physical exercise and depression among college students (the chain mediating model is shown in Figure 1).

## 2 Research objects and methods

### 2.1 Research objects

Sample size estimation: The sample size was estimated according to the formula:


$$N=\frac{Z^{2}[P(1-P)]}{E^{2}}$$

where *N* is the sample size, *Z* is the critical value of the *Z*-score for a two-tailed test when *α* = 0.05, *P* is the probability value, and *E* is the allowable error (Arya et al., 2012). In this study, *Z* = 1.96, *E* = 3%, and *p* = 28.4%. Previous research results have pointed out that the detection rate of depression among Chinese college students is 28.4% (Gao et al., 2020), resulting in a sample size of 868. Considering 10% invalid questionnaires, the final required sample size is 965.

This study adopted a convenience sampling method to conduct an online questionnaire survey among students from 8 universities in 5 cities in Henan Province (Zhengzhou, Luoyang, Pingdingshan, Xinxiang, Xinyang). Before the survey, the content and purpose of the survey were explained to each participant, and their informed consent was obtained. The questionnaires were distributed by professionally trained teachers through class WeChat groups. Participants were also informed that they could withdraw at any time if they felt uncomfortable. All materials and procedures of this study have been approved by the Ethics Committee of China University of Geosciences, and all research procedures were conducted in accordance with the ethical principles in the Declaration of Helsinki. Anonymity and confidentiality of participants were guaranteed, and participation was completely voluntary. A total of 2,120 students participated in the survey. After excluding invalid questionnaires due to excessively short response times and consistent answers, 2,021 valid questionnaires were retained, with a response rate of 95.33%. Among them, there were 745 males (36.86%) and 1,276 females (63.14%); 958 freshmen (47.40%), 774 sophomores (38.30%), 233 juniors (11.53%), 31 seniors (1.53%), and 25 graduate students (1.24%); 951 students majoring in liberal arts (47.06%), 716 in science (35.43%), 77 in physical education (3.81%), and 277 in art (13.71%); 307 students from urban families (15.19%) and 1,714 from rural families (84.81%); 184 only children (9.10%) and 1,837 non-only children (91.90%); the average age of the subjects was 19.02 ± 1.38 years old.

### 2.2 Research tools

#### 2.2.1 Physical activity rating scale-3 (PARS-3)

The Physical Activity Rating Scale-3 (PARS-3), revised by Liang (1994), was used to evaluate the physical exercise level of the subjects. This scale measures the intensity, time, and frequency of physical activity of the subjects in the past month, with a total of 3 items, each with five options, divided into 5 levels with a 1–5 scoring system. Exercise volume = intensity × (time - 1) × frequency. The minimum score of exercise volume is 0, and the maximum is 100. The Cronbach’s *α* coefficient of this scale in this study was 0.74.

#### 2.2.2 Center for epidemiologic studies depression scale (CES-D)

The Center for Epidemiologic Studies Depression Scale (CES-D) compiled by Radloff and revised by Zhang et al. (2010) was used to evaluate the depressive symptoms of the subjects. This scale consists of 20 items in 4 dimensions: depressive mood, positive mood, physical symptoms, and interpersonal problems. A 0–3 scoring system was adopted, where 0 represents “less than 1 day”, 1 represents “1–2 days”, 2 represents “3–4 days”, and 3 represents “5–7 days.” The total score of the scale ranges from 0 to 60, with higher scores indicating higher levels of depression in the subjects. The Cronbach’s *α* coefficient of this scale in this study was 0.89.

#### 2.2.3 General self-efficacy scale (GSES)

The General Self-Efficacy Scale (GSES), translated and revised by Wang et al. (2001), was used. This scale consists of 10 items, with a 1–4 scoring system, from 1 representing “completely incorrect” to 4 representing “completely correct.” The total score of the scale ranges from 10 to 40, with higher scores indicating higher levels of self-efficacy in the subjects. The Cronbach’s *α* coefficient of this scale in this study was 0.95.

#### 2.2.4 Self-identity scale (SIS)

The Self-Identity Scale (SIS) compiled by Ochse and Plug (1986) was used. This scale consists of 19 items in 4 dimensions: self-role identity, self-value identity, self-identity change, and self-motivation identity. A 1–4 scoring system was adopted, from 1 representing “completely inconsistent” to 4 representing “very consistent.” The total score of the scale ranges from 19 to 76, with higher total scores indicating better self-identity of the subjects. The Cronbach’s *α* coefficient of this scale in this study was 0.76.

### 2.3 Statistical processing

This study mainly used SPSS 27.0 software for data processing. The data processing involved the following steps: (1) To test for common method bias, all measurement items were subjected to unrotated exploratory factor analysis, and Harman’s single-factor test was used for analysis. (2) Independent sample *t*-test and one-way analysis of variance (ANOVA) were used to test the differences of each variable in demographic characteristics. (3) SPSS 27.0 software was used for descriptive statistics of the valid data collected in this study, and the correlation between variables was analyzed. (4) The SPSS PROCESS 4.2 macro developed by Hayes (2013) was used for multiple mediating effect analysis. This analysis tested the direct association between physical exercise and depression among college students, as well as the statistical chain mediating effect of self-efficacy and self-identity between the two variables. Notably, the sequential order of self-efficacy and self-identity is based on theoretical assumptions rather than empirical verification. Subsequently, the Bootstrap method was used to test the significance of the statistical associations in different paths.

## 3 Research results

### 3.1 Common method bias test

Participants responded to the questionnaire through self-report, which may lead to common method bias. To mitigate the potential impact of common method bias on the results, the following control measures were implemented during the research process: explicitly informing participants that the data would be used solely for research purposes, ensuring the anonymity of participants, and reversing the scoring of certain items (Zhou and Long, 2004).

To further enhance the rigor of the study, Harman’s single-factor analysis was used to test for common method bias (Podsakoff et al., 2003). The results showed that there were 8 factors with eigenvalues greater than 1, and the variance explanation rate of the largest factor was 22.20%, which was less than the critical value of 40% (Tang and Wen, 2020), indicating that there was no serious common method bias in this study.

### 3.2 Comparison of scores of each variable in demographic characteristics

Table 1 presents the differences in physical exercise, self-efficacy, self-identity, and depression across key demographic characteristics. The results of independent sample t-tests and one-way ANOVA showed statistically significant differences in core variables among subgroups (*p* < 0.01 or *p* < 0.001): (1) Gender and only-child status: Males and only children scored significantly higher in physical exercise and self-efficacy than females and non-only children, respectively; (2) Age and grade: Students aged ≥22 years, seniors, and graduate students had higher physical exercise and self-efficacy scores; freshmen showed higher self-identity and lower depression scores compared to sophomores and juniors; (3) Major: Physical education majors scored the highest in physical exercise and self-efficacy, while art majors had the highest depression scores. These subgroup differences justify the inclusion of gender, age, grade, major, and only-child status as control variables in the subsequent mediation analysis, ensuring that the observed mediating associations are not confounded by demographic factors.

**Table 1:** Comparison of scores of physical exercise, self-efficacy, self-identity, and depression in different demographic characteristics (M ± SD) (*n* = 2,021).

Demographic variable	Category	Physical exercise	Self-efficacy	Self-identity	Depression
Gender	Male	20.01 ± 21.93	25.44 ± 6.74	54.23 ± 6.59	11.91 ± 9.08
	Female	8.91 ± 12.38	24.14 ± 5.77	54.45 ± 6.00	12.14 ± 8.75
	*t*	12.69***	4.42***	−0.76	−0.57
Only child	Yes	17.38 ± 20.58	26.24 ± 6.80	54.60 ± 7.03	12.16 ± 10.24
	No	12.57 ± 16.98	24.46 ± 6.09	54.34 ± 6.14	12.04 ± 8.72
	*t*	3.07**	3.43***	0.47	0.15
Household registration	Rural	12.84 ± 17.16	24.60 ± 6.12	54.42 ± 6.15	11.94 ± 8.74
	Urban	13.92 ± 18.65	24.71 ± 6.50	54.07 ± 6.61	12.69 ± 9.55
	*t*	−1.00	−0.29	0.90	−1.36
Age	≤17 years old	12.25 ± 16.49	24.96 ± 6.71	55.28 ± 6.41	12.52 ± 8.55
	18–21 years old	12.61 ± 16.87	24.53 ± 6.12	54.32 ± 6.20	12.02 ± 8.86
	≥22 years old	29.76 ± 28.01	27.32 ± 6.73	54.37 ± 6.50	12.35 ± 9.83
	*F*	23.83***	5.05**	1.05	0.16
Grade	Freshman	13.86 ± 17.22	24.98 ± 6.07	55.07 ± 6.45	10.77 ± 8.39
	Sophomore	10.57 ± 14.92	23.72 ± 6.05	53.66 ± 5.62	13.32 ± 8.82
	Junior	14.67 ± 20.87	25.61 ± 6.50	53.99 ± 6.83	12.94 ± 10.30
	Senior	23.32 ± 28.43	27.35 ± 7.18	54.94 ± 6.77	11.90 ± 8.25
	Graduate student	27.52 ± 25.31	26.32 ± 5.91	52.44 ± 5.59	13.80 ± 8.41
	*F*	12.25***	8.56***	6.53***	9.97***
Major	Liberal arts	10.26 ± 13.64	24.21 ± 5.68	54.74 ± 6.07	11.69 ± 8.76
	Science	14.97 ± 17.67	25.13 ± 6.41	54.11 ± 6.25	12.14 ± 8.78
	Physical education	37.73 ± 32.22	28.35 ± 6.90	55.55 ± 6.95	9.91 ± 7.93
	Art	10.49 ± 16.32	23.67 ± 6.55	53.45 ± 6.32	13.68 ± 9.51
	*F*	71.50***	14.86***	4.46**	5.17**

*p < 0.05, **p < 0.01, ***p < 0.001. The same below.

### 3.3 Descriptive statistics and correlation analysis

Table 2 presents the means, standard deviations, and Pearson product–moment correlation matrices of the four core variables (physical exercise, depression, self-efficacy, and self-identity). The results of correlation analysis showed that there were significant correlations between physical exercise, depression, self-efficacy, and self-identity. Physical exercise was negatively correlated with depression (*r* = −0.188, *p* < 0.01), positively correlated with self-efficacy (*r* = 0.223, *p* < 0.01), and positively correlated with self-identity (*r* = 0.150, *p* < 0.01). Depression was negatively correlated with self-efficacy (*r* = −0.269, *p* < 0.01) and negatively correlated with self-identity (*r* = −0.621, *p* < 0.01). Self-efficacy was positively correlated with self-identity (*r* = 0.311, *p* < 0.01).

**Table 2:** Means, standard deviations, and correlation coefficients of each research variable (*n* = 2,021).

Variable	*M*	*SD*	1	2	3
1. Physical exercise	13.01	17.39	—		
2. Depression	12.05	8.87	−0.188**	—	
3. Self-efficacy	24.62	6.18	0.223**	−0.269**	—
4. Self-identity	54.37	6.22	0.150**	−0.621**	0.311**

#### 3.3.1 Multicollinearity test

Since there was a significant correlation among all variables, there might be a multicollinearity issue, which could lead to unstable results. Therefore, this study conducted a multicollinearity diagnosis with depression as the dependent variable and physical exercise, self-efficacy, and self-identity as independent variables. The results showed that the tolerance values of all independent variables ranged from 0.87 to 0.94 (all greater than 0.1), and the variance inflation factors (VIF) ranged from 1.06 to 1.15 (all less than 5). This indicates that there is no obvious multicollinearity in the data of this study.

### 3.4 Chain mediating effect analysis of self-efficacy and self-identity

Model 6 in the PROCESS 4.2 plug-in of SPSS was used for mediating effect analysis, and all variables were standardized. Firstly, variables with statistically significant differences in Table 1 (gender, age, grade, major, and only child status) were used as control variables, and no mediating variables were added to test the direct path of physical exercise on depression. The test results found that before adding mediating variables, the direct path of physical exercise on depression was significant (*β* = −0.223, *t* = −9.470, *p* < 0.001), indicating that physical exercise was significantly negatively associated with depression among college students, and Hypothesis H1 was supported.

**Figure 2:**
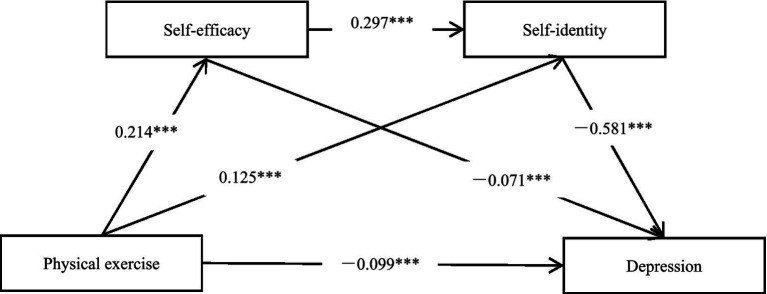
Chain mediating model.

Physical exercise was used as the predictor variable, depression as the outcome variable, and self-efficacy and self-identity as mediating variables for chain mediating analysis. As shown in Table 3 and Figure 2, all paths in the model reached a significant level (*p* < 0.001). In the direct path of physical exercise on depression, physical exercise was significantly negatively associated with depression (*β* = −0.099, *t* = −5.143, 95% CI = [−0.137, −0.061]); in the indirect association via self-efficacy, physical exercise was significantly positively associated with self-efficacy (*β* = 0.214, *t* = 9.089, 95% CI = [0.168, 0.260]), and self-efficacy was significantly negatively associated with depression (*β* = −0.071, *t* = −3.828, 95% CI = [−0.107, −0.035]); in the indirect association via self-identity, physical exercise was significantly positively associated with self-identity (*β* = 0.125, *t* = 5.387, 95% CI = [0.079, 0.170]), and self-identity was significantly negatively associated with depression (*β* = −0.581, *t* = −31.552, 95% CI = [−0.617, −0.545]); in the sequential association through the theoretically assumed path of self-efficacy → self-identity, self-efficacy was significantly positively associated with self-identity (*β* = 0.297, *t* = 13.810, 95% CI = [0.255, 0.339]).

**Table 3:** Regression analysis of variable relationships in the chain mediating model.

Outcome variable	Predictor variable	*R*	*R^2^*	*F*	*β*	*t*
Depression		0.230	0.053	18.697***			Gender				−0.055	−2.360*	Age				0.003	0.088	Grade				0.102	2.995**	Major				0.050	2.208*	Only child status				−0.007	−0.304	Physical exercise				−0.223	−9.470***
	Self-efficacy		0.236	0.056	19.741***			Gender				−0.032	−1.392	Age				0.026	0.785	Grade				−0.012	−0.353	Major				−0.017	−0.765	Only child status				−0.063	−2.913**	Physical exercise				0.214	9.089***
		Self-identity		0.350	0.123	40.269***			Gender				0.082	3.659***	Age				−0.065	−2.011*	Grade				−0.040	−1.217	Major				−0.045	−2.070*	Only child status				0.005	0.236	Physical exercise				0.125	5.387***	Self-efficacy				0.297	13.810***
			Depression		0.635	0.404	170.215***			Gender				−0.015	−0.820	Age				−0.028	−1.063	Grade				0.076	2.808**	Major				0.019	1.091	Only child status				−0.019	−1.105	Physical exercise				−0.099	−5.143***	Self-efficacy				−0.071	−3.828***	Self-identity				−0.581	−31.552***

The bias-corrected nonparametric percentile Bootstrap method (5,000 resamplings) was used to test the significance level of the multiple mediating effects of the model. If the 95% confidence interval of the mediating effect path does not contain 0, it indicates that the mediating effect of the mediating path is significant, and the mediating path is composed of the indirect effects generated by the paths (see Table 4). Firstly, there is a significant indirect statistical association between physical exercise and depression via self-efficacy (*β* = −0.015, 95% CI = [−0.024, −0.006]); secondly, a significant indirect statistical association exists between physical exercise and depression via self-identity (*β* = −0.072, 95% CI = [−0.104, −0.042]); thirdly, there is a significant sequential statistical association between physical exercise and depression through the theoretically assumed path of self-efficacy → self-identity (*β* = −0.037, 95% CI = [−0.049, −0.026]). None of the 95% confidence intervals for these three indirect associations include 0, indicating statistical significance. The total value of these indirect statistical associations is (−0.015) + (−0.072) + (−0.037) = −0.124, with the total association value between physical exercise and depression being −0.223. The proportions of each indirect statistical association relative to the total association are 6.73%, 32.29%, and 16.59%, respectively. These results suggest that the indirect association via self-identity was larger than that via self-efficacy, and the total proportion of indirect statistical associations accounts for 55.61%, which is higher than the direct effect, indicating that the overall indirect associations appear to have practical relevance in the model, although the practical contribution of the self-efficacy pathway is comparatively modest relative to self-identity and the sequential pathway. The results indicate that the statistical mediating and statistical chain mediating associations proposed in Hypotheses 2, 3, and 4 are all statistically significant.

**Table 4:** Test of mediating effects based on the bootstrap method.

Effect type	Path relationship	Effect value	SE	Effect size	95% confidence interval	
					*LLCI*	*ULCI*
Direct effect	PARS-3 → CES-D	−0.099	0.019	44.39%	−0.137	−0.061
Mediating effect	PARS-3 → GSES→CES-D	−0.015	0.005	6.73%	−0.024	−0.006
	PARS-3 → SIS→CES-D	−0.072	0.016	32.29%	−0.104	−0.042
	PARS-3 → GSES→SIS→CES-D	−0.037	0.006	16.59%	−0.049	−0.026
Total mediating effect		−0.124	0.017	55.61%	−0.158	−0.092
Total effect		−0.223	0.024		−0.270	−0.177

PARS-3, Physical exercise; GSES, Self-efficacy; SIS, Self-identity; CES-D, Depression.

## 4 Discussion

### 4.1 The association between physical exercise and depression among college students

The results of this study show that physical exercise is significantly negatively correlated with depressive symptoms among college students, which supports Hypothesis H1. This conclusion is highly consistent with previous relevant research results (Wang Z. et al., 2025), further supporting that physical exercise, as a low-cost and easy-to-promote health promotion method, is associated with improvements in negative psychological states such as depression and anxiety, and holds core value for maintaining mental health, and this positive association may be long-term and sustainable (Wang and Zhang, 2025). From a theoretical perspective, this association may be understood in terms of both physiological and psychological processes. At the physiological level, regular physical exercise is associated with enhanced body metabolism and more balanced secretion of neurotransmitters such as endorphins, serotonin, and dopamine. These substances, as core carriers of emotion regulation, are associated with improved mood states and reduced physical discomfort and low mood related to depression (Gorman, 1996). At the psychological level, the attention distraction hypothesis theory holds that depressed individuals often fall into excessive rumination and self-blame about negative life events, and this negative cognitive model will continuously strengthen depressive symptoms; physical exercise is associated with reduced focus on negative thoughts by constructing specific exercise goals and immediate experiences, freeing them from the closed loop of negative cognition, and may be related to alleviated painful physical feelings accompanied by depression (Wang et al., 2021). In addition, a large-sample study of 2,374 students from 8 schools also confirmed that students who actively participate in physical exercise show a significantly lower prevalence of depressive symptoms than those who do not participate in physical exercise (Chi and Wang, 2022). College students are confronted with multiple psychological stressors, such as academic pressure and social adaptation challenges. These stress factors are likely to induce or exacerbate depressive emotions. Physical exercise may serve as a convenient and accessible way to regulate emotions. Whether in the form of group sports or individual exercise on campus, physical exercise may provide a convenient context for college students to regulate psychological stress and emotional states, and may be related to lower depressive symptoms.

### 4.2 The mediating association of self-efficacy in the relationship between physical exercise and depression among college students

This study found that self-efficacy is significantly negatively associated with depression among college students. At the same time, physical exercise is not only directly correlated with college students’ depression levels but also shows an indirect association with depression through the intermediate path of self-efficacy. Hypothesis H2 was statistically supported, indicating that self-efficacy forms a significant but relatively modest mediating association between physical exercise and depression among college students. This result is consistent with relevant research conclusions. Physical exercise is significantly positively correlated with self-efficacy (Wang et al., 2022), and self-efficacy may be regarded as a psychological factor related to lower depressive symptoms (Pu et al., 2016). Together, these findings suggest that self-efficacy may be relevant to mental health among college students. The possible reason is that regular physical exercise is associated with enhanced self-confidence, self-cognition, and self-worth, is associated with fewer negative emotions and may contribute to a better emotional state. This positive experience may strengthen their recognition of their own abilities and may be related to higher levels of self-efficacy. In addition, individuals with high self-efficacy often have good emotional regulation abilities (Yang et al., 2022) and hold positive self-cognition, and are more inclined to regard external setbacks and difficulties as temporary challenges, which is associated with fewer depressive emotions related to psychological pressure (Li, 2019). It is worth noting that previous intervention studies have suggested that self-efficacy is a modifiable psychological characteristic (Luthans et al., 2010). Therefore, the observed association between physical exercise and self-efficacy may provide a cautious practical reference for mental health promotion in university settings. Accordingly, self-efficacy should be interpreted as a secondary and supportive indirect pathway rather than a dominant mediator in the relationship between physical exercise and depression.

Although the results of this study indicate that the indirect statistical association via self-efficacy is statistically significant (*β* = −0.015, accounting for 6.73% of the total association), its practical magnitude is relatively small compared to the other two paths. This suggests that while self-efficacy serves as a statistically valid mediating link between physical exercise and depression, its independent association with lower depressive tendencies is relatively modest. The relatively small effect may be attributed to the fact that self-efficacy primarily reflects short-term confidence in task completion, while depression among college students is often influenced by more stable psychological factors. Nevertheless, the statistical significance of this path still supports the theoretical logic of social cognitive theory, suggesting a possible link between physical exercise and self-related beliefs.

### 4.3 The mediating association of self-identity in the relationship between physical exercise and depression among college students

The results of this study show that, in addition to self-efficacy, self-identity also forms a significant mediating association between physical exercise and depression among college students, and Hypothesis H3 is established. As the core confirmation of an individual’s self-worth and life direction, the stability of self-identity is associated with reduced vulnerability to the impact of external stress on emotions, which is consistent with Erikson’s identity development theory (Ochse and Plug, 1986). Therefore, the indirect association via self-identity appears stronger than that via self-efficacy, the latter focusing more on the short-term regulatory role of “belief in ability”. From the perspective of the relationship between physical exercise and self-identity, regular physical exercise not only provides a unique communication platform for college students to interact with others frequently but also fosters good social support (Zhang, 2008), providing an important context for college students to explore themselves and confirm their values. At the same time, in the process of continuous participation in exercise, individuals can continuously accumulate a clear cognition of “self-ability” and “self-worth” by breaking through exercise challenges, improving physical fitness, or clarifying their own roles and contributions in team sports, which is correlated with the gradual development of self-identity (Salmon, 2001), and may help college students develop clearer self-positioning and greater self-acceptance. From the perspective of the relationship between self-identity and depression, higher levels of self-identity are associated with a lower likelihood of depressive symptoms (Wang L. et al., 2025). This may be because individuals with stable self-identity have clearer cognition and a more accepting attitude towards themselves. When facing negative events such as academic pressure and interpersonal conflicts, they are less likely to have self-doubt or value negation due to external evaluations, thereby reducing the breeding of negative emotions such as depression and anxiety. In addition, individuals with high self-identity have a high level of psychological resilience (Zhao, 2020), which is associated with better emotional stability when encountering setbacks and a lower likelihood of negative emotions evolving into depressive symptoms (Zhang et al., 2024).

### 4.4 The sequential mediating association of self-efficacy and self-identity in the relationship between physical exercise and depression among college students

The results further suggested a significant sequential indirect association involving self-efficacy and self-identity in the relationship between physical exercise and depression among college students (*β* = −0.037, accounting for 16.59% of the total association), supporting Hypothesis H4. When comparing the relative magnitudes of the three indirect paths, the independent mediating effect of self-identity is the strongest (32.29%), followed by the chain mediating effect (16.59%), while the independent mediating effect of self-efficacy is the smallest (6.73%). This pattern indicates that stable self-identity appears to show a stronger indirect association in the relationship between physical exercise and depression, while the sequential path of “physical exercise → self-efficacy → self-identity” adds explanatory value to the overall association pattern by integrating short-term belief enhancement and long-term self-confirmation. From the perspective of social cognitive theory, there is a dynamic interaction between individuals’ beliefs, motivations, and other subjective psychological factors and behaviors and emotional reactions. Subjective factors guide behavioral choices and execution, and behavioral results in turn strengthen or adjust psychological beliefs, ultimately affecting emotional experiences (Schunk and DiBenedetto, 2020). Regular physical exercise, as a positive behavioral practice, is associated with stronger beliefs in one’s own abilities—namely, self-efficacy through specific experiences such as goal achievement and skill improvement. Individuals with high self-efficacy often have stronger achievement motivation, willingness to accept challenges, and psychological resilience. In the continuous process of self-exploration and practice, they are more likely to clearly perceive their own value, clarify their self-positioning, and thus gradually build a stable and positive self-identity (Liu and Cheng, 2017). Finally, higher levels of self-identity are associated with clearer self-cognition and greater self-acceptance among college students. When facing negative events such as academic pressure and interpersonal conflicts, they tend to demonstrate more effective emotional regulation and stress-coping skills, which are associated with reduced negative emotions and better psychological resilience, thereby showing a tendency toward fewer depressive emotions.

### 4.5 Research significance

This study identifies significant associative patterns of physical exercise, self-efficacy, and self-identity on college students’ depression, clarifying that physical exercise is not only directly correlated with lower depressive symptoms among college students but also shows an indirect association with reduced depressive tendencies through two separate mediating paths (positive associations with self-efficacy and self-identity) and the chain mediating association path of “physical exercise → self-efficacy → self-identity”. Notably, the relative magnitudes of these paths vary: self-identity’s independent mediating effect is practically more meaningful, while self-efficacy’s independent effect is statistically significant but relatively modest in practical terms. This distinction may provide cautious implications for university mental health promotion. However, these practical implications should be interpreted with caution, because the present sample was drawn from universities in Henan Province and showed demographic imbalance, particularly in terms of gender and rural family background. Therefore, the applicability of these findings to other populations, urban environments, and geographic regions remains limited and requires further verification. In particular, colleges and universities may consider paying greater attention to students’ self-identity, while interpreting self-efficacy as a secondary and supportive pathway whose practical contribution appears comparatively modest. In addition, from a practical perspective, colleges and universities may consider integrating physical exercise into mental health education and designing targeted exercise programs based on students’ demographic characteristics (such as gender, major, and grade). At the same time, they may consider lowering the threshold for students to participate in physical exercise and stimulating their exercise initiative by optimizing physical education curriculum settings, improving campus sports facilities, and organizing campus sports and cultural activities. They may also consider setting hierarchical exercise goals in physical education teaching, providing timely feedback on exercise results, helping students accumulate successful experiences associated with higher self-efficacy, and carrying out self-cognition exploration activities through themed class meetings and group psychological counseling to guide students in developing a stable and positive self-identity.

### 4.6 Limitations and future directions

Although this study identified a significant sequential indirect association involving self-efficacy and self-identity between physical exercise and depression among college students, there are still some limitations. In particular, the total indirect effect (55.61%) generated through the independent mediating roles of self-efficacy and self-identity, as well as their chain mediating role, accounts for a relatively large proportion of the total effect. The possible reasons for this phenomenon are as follows: First, this study adopts a cross-sectional design, which cannot definitively establish causal relationships, and all variables are measured by self-report scales, which may have potential impacts on the mediating results. Although Harman’s single-factor test indicated no serious common method bias, participants may still be affected by subjective factors such as social desirability bias and consistent response tendencies, which may slightly overestimate the strength of associations between variables, one of the reasons for the relatively large total indirect effect. Future research can integrate objective measurement tools, such as objective physical activity measures or clinician-rated mental health indicators, and adopt a longitudinal follow-up design to explore whether the relationships between variables change over time. This will enhance the objectivity of the results and provide more robust support for potential causal inferences. Second, this study is mainly based on individual-level variables, and there may be unconsidered psychosocial factors. Future research will incorporate social-level variables, such as exploring the potential impacts of social support and family functioning. In addition, although self-efficacy and self-identity were treated as theoretically distinct constructs, both reflect aspects of positive self-cognition and may show partial conceptual and measurement overlap. In the present study, self-efficacy and self-identity were moderately correlated (*r* = 0.311), and no severe multicollinearity was observed (VIF = 1.06–1.15). However, we did not conduct an additional discriminant validity test, such as confirmatory factor analysis comparing one-factor and two-factor models. Therefore, the empirical distinctiveness of these two constructs should be interpreted with caution. This construct overlap may have amplified the association strength of the chain mediating pathway, thereby contributing to an overestimation of the total indirect effect. Future research is encouraged to formally test the discriminant validity of the GSES and SIS using item-level measurement models. Finally, 63.14% of the samples in this study are female, and the participants are mainly from Henan Province. Due to geographical constraints, 84.81% of the participants are from rural families. The homogeneity of these demographic characteristics or regional cultural specificity may strengthen the associations between physical exercise, self-related constructs, and depression, thereby increasing the effect size. It must be emphasized that this relatively large indirect effect does not imply a causal relationship, but rather reflects the cumulative association strength between variables in the current sample. Therefore, to enhance external validity and theoretical generalization, future research should expand the sample scope to include different regions, different types of universities, and a more balanced distribution of gender and family backgrounds. At the same time, multi-source data collection methods should be adopted to further control the potential impacts of common method bias and construct overlap, so as to improve the robustness and generalizability of the research results.

## 5 Conclusion

This study examined the association between physical exercise and depression among college students and identified statistically significant indirect associations through self-efficacy, self-identity, and their sequential pathway. These findings enrich the literature on physical exercise and mental health in college populations and may provide cautious practical implications for university mental health promotion. However, because the sample was drawn from universities in Henan Province and showed demographic imbalance, the findings should be generalized to other populations, urban environments, and geographic regions with caution. Future studies based on more diverse and balanced samples are needed to further examine the generalizability of these findings.

## Data Availability

The raw data supporting the conclusions of this article will be made available by the authors, without undue reservation.
